# Supplementary material for: Study protocol for RUFUS—A randomized mixed methods pilot clinical trial investigating the relevance and feasibility of rumination-focused cognitive behavioral therapy in the treatment of patients with emergent psychosis spectrum disorders
Source: PLoS One. 2024 Jan 25;19(1):e0297118. doi: 10.1371/journal.pone.0297118 (PMC10810475; doi:10.1371/journal.pone.0297118)
Supplement: S2 Checklist — (DOCX) [file pone.0297118.s002.docx]

SPIRIT

| Section/item | ItemNo | Description |
| --- | --- | --- |
| Title | 1 | Study protocol for RUFUS - a randomized mixed methods pilot clinical trial investigating the relevance and feasibility of rumination-focused cognitive behavioral therapy in the treatment of patients with emergent psychosis spectrum disorders |
| Trial registration | 2a | Clinicaltrials NCT05851950 |
| Protocol version | 3 | 1.0 |
| Funding | 4 | The study received no funding |
| Roles and  responsibilities | 5a | Lars Clemmensen^1*^, Christin Nymann Lund^2^, Birgitte S. Andresen^3^, Julie Midtgaard^2,4^, Louise Birkedal Glenthøj^1,5^.  ^1^VIRTU Research Group, Copenhagen Research Center on Mental Health (CORE), Copenhagen University Hospital, Denmark. ^2^Center for applied research in mental health care (CARMEN), Mental Health Center Glostrup, ^3^Mental Health Center Glostrup, ^4^Department of Clinical Medicine, University of Copenhagen, ^5^Department of Psychology, University of Copenhagen, Copenhagen, Denmark  CNL, LBG, and JM developed the study/drafted the protocol. LC wrote the main manuscript text. All authors read and approved the final manuscript. |
| **Introduction** | | |
| Background and rationale | 6a | See “background” section of the ms |
| Objectives | 7 | The aim of the current study is to investigate the feasibility and acceptability of group RFCBT in the treatment of young people with psychosis spectrum disorders as well as investigating potential indications of treatment efficacy. |
| Trial design | 8 | The study is a mixed methods pilot RCT combining quantitative and qualitative methods. The study will enroll a total of 60 patients from Mental Health Center Glostrup, Mental Health Services in Capital Region of Denmark, which will be randomly assigned to one of the two arms: 1. Experimental group (13 weeks of standard (OPUS) treatment and group RFCBT) or 2. Control group (13 weeks of standard treatment (OPUS)). Patients are assessed at baseline and at treatment cessation (see figure 1). Following completion of post-treatment assessments, the patients in the control group will be offered RFCBT. The study is fulfilling the CONSORT criteria for non-pharmacological treatments and the extension to randomized pilot and feasibility trials. |
| **Methods: Participants, interventions, and outcomes** | | |
| Study setting | 9 | The study is carried out within the framework of OPUS. Mental Health Center Glostrup, Mental Health Services in Capital Region of Denmark |
| Eligibility criteria | 10 | Inclusion criteria  1. Age 18-35  2. Diagnosed with a psychosis spectrum disorder (ICD-10 F2x)  3. At least 8 months left of their OPUS treatment  4. The presence of rumination behavior as assessed by a score of minimum 30 on the Perseverative Thinking Questionnaire (PTQ)  5. Danish-speaking  Exclusion criteria  1. Substance abuse or positive symptoms that make participation in therapy difficult  2. Severe suicidal thoughts/behavior  3. Not capable of providing informed consent  4. Mild, moderate, or severe intellectual disability (IQ ˂ 70)  5. Planned adjustment of antidepressant and/or antipsychotic treatment (noted in the patient's medical record) |
| Interventions | 11a | Experimental group  The experimental intervention consists of group-based, manualized RFCBT that follows the principal treatment components outlined in the treatment manual developed and published by Watkins (Watkins & Baracaia, 2001). The manual has been and translated for use in a Danish context (Kistrup et al., 2017). The treatment comprises 11 groups-session and two individual sessions and is expected to last approximately three months. The treatment is offered as an add on to standard OPUS treatment (2-year intensive outpatient treatment). Initially, an individual group preparatory session is conducted (one hour) followed by 11 group sessions of 2 hours duration taking place once a week. The intervention ends with an individual session (duration of one hour). The therapy includes review and completion of hand-outs, psychoeducation, or dissemination of important messages from the manual, practical exercises and behavioral experiments (patients cooperate in pairs), guided visualization exercises as well as dialogue and exchange of experiences. A primary goal is to acquire skills so that one does not automatically fall into the habit of ruminating or avoiding. The intervention takes place in OPUS and is overseen by an experienced psychologist (experiment leader) and a co-therapist.  Control group  Patients in the control group receive standard OPUS treatment and are offered the experimental intervention on completion of follow-up assessment (expected at 3 months). OPUS treatment is handled by an interdisciplinary OPUS team and consists primarily of medical treatment, psychoeducation, training in symptom management and social skills as well as family involvement/ treatment. All patients in OPUS have a contact doctor and a contact person who is responsible for coordinating the treatment and collaborating with municipal bodies. |
|  | 11b | Side effects and adverse events will be monitored and recorded throughout the study period. |
| Outcomes | 12 | see section on “outcomes” in the ms |
| Participant timeline | 13 | see Figure 1 |
| Sample size | 14 | As the overall purpose of the study is to uncover acceptability, feasibility and provide indications of a possible treatment effect. the number of subjects is not based on a calculation of statistical power, but rather on an expectation of how many will be able to include over a project period of 12 months A total of 30 patients receiving the intervention will be compared to 30 receiving standard treatment. A sample size of 30 participants is considered adequate for a pilot-study assessing feasibility, acceptability, and providing indications of efficacy of an intervention (Bell et al., 2018). |
| Recruitment | 15 | The study is situated at Mental Health Center Glostrup, which has approximately 300 active OPUS patients divided in three teams. In previous trials with group-based CBT in OPUS, it has been possible to recruit a minimum of 10% of the total OPUS sample, thus it is realistic to include the 60 patients in a project period of 18-months |
| **Methods: Assignment of interventions (for controlled trials)** | | |
| Allocation: |  |  |
| Sequence generation | 16a | The randomization is done via the computerized randomization function in Research Electronic Data Capture (REDCap) (Harris et al., 2009) tool in ratio 1:1 on the basis of an uploaded block randomization list (block size 4-6) generated by an external party. . In addition, stratification is used to ensure equal distribution of gender between the groups. |
| Blinding (masking) | 17a | Assessors will be blinded |
| Methods: Data collection, management, and analysis | | |
| Data collection  methods | 18a Plans for assessment and collection of outcome, baseline, and other trial data, including any related processes to promote data quality (eg, duplicate measurements, training of assessors) and a description of study instruments (eg, questionnaires, laboratory tests) along with their reliability and validity, | Questionnaires and measurements are completed at baseline and repeated at treatment cessation after approximately three months. Follow-up clinician-administered measurements are conducted by investigators blind to group allocation. Patients will be instructed not to disclose their allocation prior to post-treatment assessments. In case an assessor is unblinded, the assessment will be conducted by another assessor. The assessors will be psychology master students. All assessors will receiving adequate training prior to conducting the assessments. The assessors will attend regular inter-rater reliability training on key measures in the battery. Quantitative data are collected in REDCap (Harris et al., 2009) and qualitative data are audiorecorded on a dictaphone and subsequently transcribed ad verbatim and anonymized. |
| Data  management | 19 | Quantitative data are collected in REDCap and qualitative data are audiorecorded on a dictaphone and subsequently transcribed ad verbatim and anonymized. |
| Statistical  methods | 20a | Treatment effect will be analyzed as changes in outcome measures from baseline to post-treatment in the RFCBT group using paired samples t-tests or a corresponding non-parametric test such as Pearson's chi-square test. Continuous data are presented as mean ± standard deviation (SD). A two-sided significance level of p <0.05 is used |
| **Methods: Monitoring** | | |
| Harms | 22 | Side effects and adverse events will be monitored and recorded throughout the study period. Any adverse events will be reported to the Committee on Health Research Ethics of the Capital Region Denmark |
| **Ethics and dissemination** | | |
| Consent or assent | 26a | All patients will be asked to give informed consent after oral and written information about the trial |
| Confidentiality | 27 | All personal information will be entered directly into REDCap |
| Declaration of  interests | 28 | The authors have declared that no competing interests exist. |
| Access to data | 29 | Only researchers in the study group will have access to the final trial dataset |
| Ancillary and  post-trial care | 30 | The study is covered by the Danish patient compensation |
