# Supplementary material for: Study protocol for RUFUS—A randomized mixed methods pilot clinical trial investigating the relevance and feasibility of rumination-focused cognitive behavioral therapy in the treatment of patients with emergent psychosis spectrum disorders
Source: PLoS One. 2024 Jan 25;19(1):e0297118. doi: 10.1371/journal.pone.0297118 (PMC10810475; doi:10.1371/journal.pone.0297118)
Supplement: S2 File — (DOCX) [file pone.0297118.s004.docx]

Louise Birkedal Glenthøj

University of Copenhagen, Department of Psychology Copenhagen Research Center on Mental Health (CORE) Gentofte hospitalsvej 15, 4th floor

2900 Hellerup

The Scientific Ethics Committees

Borgervænget 3, stuen 2100 København Ø

Telephone 3866 6395

Mail vek@regionh.dk

Journal no.: H-23004478 Date: 03-05-2023

**H-23004478 - RUFUS: A randomised, mixed methods pilot trial aimed at describing the relevance and feasibility of rumination-focused cognitive behavioral therapy in the treatment of patients with onset of psychosis spectrum disorder**

**Final approval.**

**The decision has been made pursuant to Executive Order No. 1338 of 1 September 2020 - Act on scientific ethical treatment of health science research projects and health data science research projects.**

I confirm receipt of the email of 28 April 2023 in response to the decision of 24 April 2023, in which conditions were set for the approval of the project.

The conditions for approval are deemed to have been met. The project is thus finally approved.

**The approval is valid until 1 August 2025** and includes the following documents:

• Trial protocol, version 1, of 24 April 2023

•Participant information, version 1, dated 24 April 2023

• Informed consent, received 18 January 2023 (file name: Sam-

thick_V1_160123)

• Questionnaires approved for use in the trial:

o BRIEF

o SFS

o PTQ

o RRS(002)

The approval applies to the notified trial sites and the notified trial manager in Denmark.

Center for Health

The committee is not the competent authority for the set of regulations on data protection. The committee assumes that the project is carried out in accordance with the data protection regulation and the data protection act.

Implementation of the project in violation of the approval can be punished with a fine or imprisonment, cf. Section 41 of the Committees Act.

**Changes**

If significant changes are made to the protocol material during the implementation of the project, these must be notified to the committee in the form of additional protocols. The changes may only be implemented after approval from the committee, cf. Section 27, subsection of the Committee Act. 1.

Notification of additional protocols must be done electronically at www.drvk.dk/anmeldelse with the already assigned notification number and password.

Significant changes include changes that may affect the safety of the subjects, interpretation of the scientific documentation on which the project is based and the implementation or management of the project. These can be, for example, changes in inclusion and exclusion criteria, trial design, number of subjects, trial procedures, duration of treatment, effect parameters, changes to the persons responsible for the trial or trial sites, as well as changes in the content of the written information material for the trial subjects.

Where new information means that the researcher is considering changing the procedure or stopping the trial, the committee must be informed of this.

**Side effects and events**

Ongoing reporting

The committee must be notified immediately if suspected serious, unexpected side effects or serious incidents occur during the project, cf. section 30, subsection of the committee act. 1. The report must be accompanied by comments on any consequences for the experiment. Only side effects and incidents occurring in Denmark must be reported. Notification must be made no later than 7 days after the sponsor or the person in charge of the trial has become aware of the incident.

When reporting, a form can be used, which can be found on the National Center for Ethics' website. The form with attachments can be submitted electronically using a digital signature.

Annual report

Once a year during the entire trial period, the committee must have sent a list of all suspected serious (expected and unexpected) side effects and serious incidents that have occurred during the trial period together with a report on the safety of the test subjects, cf. Section 30, subsection of the Committee Act . 2.

The material must be in Danish or English.

When reporting, a form must be used, which can be found on the National Center for Ethics' website. The form with attachments can be submitted electronically using a digital signature.

**Closing**

The experimenter and a possible the sponsor must notify the committee of this no later than 90 days after the end of the project, cf. section 31, subsection of the committee act. 1. The project is considered completed by the committee when the researcher has completed the collection of all information for the project.

If the project is interrupted earlier than planned, a reason for this must be sent to the committee no later than 15 days after the decision has been made, cf. Section 31, subsection of the Committees Act. 2.

If the project is not started, this and the reason for this must be communicated to the committee.

The committee requests a copy of the final research report or publication, cf. section 28, subsection of the committee act. 2. In this connection, we must draw attention to the fact that there is an obligation to publish both negative, positive and inconclusive test results, cf. Section 20, subsection of the Committees Act. 1, No. 8.

The duty to report the end of the experiment and the final report rests with the person in charge of the experiment and any joint sponsor.

**Oversight**

The committee supervises that the project is carried out in accordance with the approval, cf. sections 28 and 29 of the committee act.

**Signature on the declaration of consent**

The committee draws attention to the fact that the person in charge of the trial can delegate his duty to sign the declaration of consent to the person holding the oral information interview. In that case, there must be a written delegation to this effect at the trial site.

**Data protection - record requirements**

You must be aware that you may be obliged to have the research project signed.

If you are a researcher employed in the Capital Region, you do this by contacting Research Law in the Capital Region, which is the regional unit that administers the research register. You can read more about the list and find contact details on their website.

If you are not employed in the Capital Region, you can find information about the registration requirement in the Guidance on registration on the Danish Data Protection Authority's website.

With best regards

Marie Skovgaard Administrative employee

Copy sent to: Julie Midtgaard Klausen
