# Supplementary material for: Study protocol for RUFUS—A randomized mixed methods pilot clinical trial investigating the relevance and feasibility of rumination-focused cognitive behavioral therapy in the treatment of patients with emergent psychosis spectrum disorders
Source: PLoS One. 2024 Jan 25;19(1):e0297118. doi: 10.1371/journal.pone.0297118 (PMC10810475; doi:10.1371/journal.pone.0297118)
Supplement: S1 Protocol — (DOC) [file pone.0297118.s005.doc]

| | EXPERIMENTAL PROTOCOL  Protocol version 1, 240423  RUFUS  (Rumination-focused cognitive behavioral therapy + OPUS)    A randomised, mixed methods pilot trial targeted description of  relevance and feasibility of rumination-focused cognitive behavioral therapy  in the treatment of patients with onset psychosis spectrum disorder  **Mental health center GLOSTRUP**  OPUS Team 2nd floor  (Test site)  Christin Nymann Lund (Investigator)  Psychologist  Birgitte Stougård Andreasen  Occupational therapist  Center for Applied Research in  Mental Health Care (CARMEN)  Julie Midtgaard  Professor, psychologist, Ph.D.  **Mental health center** COPENHAGEN  VIRTU Research Group, Copenhagen Research Center on Mental health (CORE)  Louise Birkedal Glenthøj (Research manager)  Lecturer, specialist psychologist, dr. med., Ph.D. | Lars Clemmensen  Postdoc, psychologist, Ph.D.  . | | --- | --- | |
| --- | --- | --- |

# Indhold

[PROTOKOLRESUME 3](#__RefHeading___Toc124607490)

[BAGGRUND 3](#__RefHeading___Toc124607491)

[FORMÅL OG FORSKNINGSSPØRGSMÅL 5](#__RefHeading___Toc124607492)

[DESIGN 5](#__RefHeading___Toc124607493)

[SETTING 5](#__RefHeading___Toc124607494)

[POPULATION 5](#__RefHeading___Toc124607495)

[Inklusionskriterier 5](#__RefHeading___Toc124607496)

[Eksklusionskriterier 6](#__RefHeading___Toc124607497)

[STYRKEBEREGNING 6](#__RefHeading___Toc124607498)

[PROCEDURE FOR REKRUTTERING, INKLUSION OG RANDOMISERING (GRUPPEALLOKERING) 6](#__RefHeading___Toc124607499)

[INTERVENTION 7](#__RefHeading___Toc124607500)

[Venteliste-kontrolgruppe 7](#__RefHeading___Toc124607501)

[DATAINDSAMLINGSMETODER OG OUTCOMES 7](#__RefHeading___Toc124607502)

[Kvantitative metoder og outcomes 7](#__RefHeading___Toc124607503)

[Kvalitative metoder 8](#__RefHeading___Toc124607504)

[DATAANALYSE 9](#__RefHeading___Toc124607505)

[Kvantitative data 9](#__RefHeading___Toc124607506)

[Kvalitative data 9](#__RefHeading___Toc124607507)

[SIKKERHED OG ETIK 9](#__RefHeading___Toc124607508)

[Risici, bivirkninger og ulemper 9](#__RefHeading___Toc124607509)

[Behandling af personfølsomme data 9](#__RefHeading___Toc124607510)

[Videregivelse af oplysninger fra patientjournal 10](#__RefHeading___Toc124607511)

[ORGANISERING OG FINANSIERING 11](#__RefHeading___Toc124607512)

[REFERENCER 11](#__RefHeading___Toc124607513)

# PROTOCOL SUMMARY

Despite significant progress in the treatment of psychosis, including early specialized intervention for young people with onset of psychosis, known as OPUS treatment (2-year intensive outpatient treatment), psychosis spectrum disorders rank among the most debilitating and costly mental disorders. Particularly burdensome for the individual, who is typically in their early 20s when the disease breaks out, is the presence of negative symptoms (e.g. emotional exhaustion, inactivity, reduced energy, social isolation) and cognitive disturbances, which are difficult to treat effectively pharmacologically. It is also not uncommon for psychosis spectrum disorders to be accompanied by symptoms of anxiety and depression. Thus, ¾ of patients with psychosis spectrum disorders are estimated to experience symptoms of depression, and the simultaneous occurrence of psychosis and depression is a significant predictor of impaired quality of life. Rumination appears in connection with several different mental disorders, and is thus recognized as a transdiagnostic phenomenon, and may underlie psychiatric multimorbidity (psychosis, anxiety and depression). Rumination is widespread among young people with psychosis spectrum disorders and the consequence of rumination can be the initiation of avoidance behavior that can reinforce the negative consequences of the psychotic disorder and impair the patient's level of functioning. Group-based rumination-focused cognitive behavioral therapy (RFCBT) has shown a good effect in the treatment of rumination in patients with depression, but so far untested in the treatment of young people with an onset of psychosis spectrum disorder and at the same time pronounced rumination/depression/negative symptoms.

Against this background, the purpose of the present study is to describe the relevance and feasibility of RFCBT in connection with early intervention (OPUS) for young people with onset of psychosis. The study is organized as a randomised, mixed methods pilot trial. It is expected that the experiment will include approx. 60 young people in treatment for onset psychosis spectrum disorder (OPUS), who are offered a course with RFCBT (11 weekly sessions of 2 hours duration) in addition to OPUS standard treatment (intervention group). These are compared with a similar group of young people who only receive OPUS standard treatment, and are offered the intervention after 12 weeks (waiting list control group). Distribution in the intervention and waiting list control group is determined via lottery. All participants (intervention and waiting list control group) are assessed and answer questionnaires at baseline, which are repeated after 12 weeks. These focus on worry/rumination behaviour, negative symptoms, problem solving, functioning and quality of life. With reference to the purpose and design of the study (pilot trial), no distinction is made between primary and secondary outcomes. In addition, semi-structured interviews are conducted with participants in the intervention group before the start of RFCBT and at the end of RFCBT. The aim here is to describe motivation for and experience with participation. Finally, continuous registration is made of the proportion of surveyed patients who say yes to RFCBT, as well as the frequency of attendance and dropouts along the way. Through analysis/measurement of changes from baseline and after 12 weeks within the intervention group and between the groups, the results of the study will provide answers as to whether it is possible and relevant to offer RFCBT complementary to OPUS, including which outcome(s) be relevant to pursue in a possible impact study, as well as any possible individual, organizational and methodological challenges that may be associated with this.

**BACKGROUND**

In Denmark, each year between 500 and 1000 people are affected by schizophrenia and other psychotic disorders (collectively termed psychosis spectrum disorders), which cause changes in thought and behaviour. Despite significant progress in treatment, including an increased focus on early intervention (including 2-year intensive outpatient treatment, OPUS) and improved medical treatment options, psychosis spectrum disorders are still described as one of the most painful and resource-demanding mental disorders [1-3]. The disease most often develops in the late teenage years and early adulthood [1] and typically debuts in the early 20s.

Psychosis spectrum disorders are characterized by positive symptoms (delusions, thought disorders, hallucinations), negative symptoms (such as lethargy, lethargy, affect-flattening, lack of initiative, passivity and social withdrawal) and cognitive disturbances (decreased attention, poor memory and difficulty in planning) ). While there is a good effect of pharmacological treatment on positive symptoms, effective methods to reduce negative symptoms and cognitive disturbances are still missing [4]. Negative symptoms are also highlighted by the patients as particularly damaging to their quality of life. In addition, 75% of patients with psychosis spectrum disorders experience depression and 65% experience anxiety symptoms [5-10].

**OBJECTIVES AND RESEARCH QUESTIONS**

The purpose of the experiment is to uncover the relevance and feasibility of rumination-focused cognitive behavioral therapy (RFCBT) in the treatment of young people with a psychotic spectrum disorder. Through this, we will examine the basis for preparing a protocol for a larger randomized trial. Specifically, answers are sought to the following three research questions:

1. What possible changes in rumination behaviour, negative symptoms, depressive symptoms, problem solving, level of functioning and quality of life are associated with participation in RFCBT among young people in treatment for early-onset psychosis spectrum disorder?

2. How do young people with a psychosis spectrum disorder justify their desire to participate in RFCBT, and what are their experiences with this?

3. To what extent and how is it possible to recruit and retain young people with psychosis spectrum disorders for RFCBT?

**DESIGN**

With the overall aim of uncovering the relevance and feasibility of a hitherto untested intervention, the study is organized as a randomized pilot trial that combines quantitative and qualitative methods. Via lottery, the participants are allocated to an intervention group (standard treatment + RFCBT; course with 11 group sessions of 2 hours duration) or a waiting list control group (standard treatment and subsequent offer of RFCBT). The choice of design reflects the complexity of the intervention as well as the desire to establish a scientifically informed basis for a possible subsequent scaled-up randomized controlled trial.

**SETTING**

The study is carried out within the framework of OPUS. OPUS is a 2-year intensive outpatient treatment service for patients aged 18-35 with a psychosis spectrum disorder. OPUS treatment is handled by an interdisciplinary OPUS team. All patients in OPUS have a contact doctor and a contact person who is responsible for coordinating the treatment and collaborating with municipal bodies. The treatment primarily consists of medical treatment, psychoeducation, training in symptom management and social skills as well as family discussions.

**POPULATION**

The trial is aimed at young people between the ages of 18-35 referred to OPUS treatment on the basis of a first episode psychotic disorder or schizotypy. The following specific inclusion and exclusion criteria apply to the patients who can be included:

Inclusion criteria

Patients can be included if they

• is diagnosed with a psychosis spectrum disorder (ICD-10 F2x)

• have at least 8 months left of their OPUS course

• recognition of rumination behavior in the form of a minimum score of 30 in connection with completing the Perseverative Thinking Questionnaire (PTQ) [32]

• are Danish-speaking

Exclusion criteria

Patients will be excluded if they:

• has active abuse or positive symptoms that make participation in therapy difficult

• has severe suicidal thoughts/behaviour

• lacks capacity to consent

• is mentally retarded (IQ ≤70)

• planned adjustment of antidepressant and/or antipsychotic treatment (noted in the patient's medical record)

**POWER CALCULATION**

With reference to the overall purpose of the study to uncover relevance and feasibility rather than effect, which i.a. is justified by the absence of previous studies in the area, the number of subjects is not based on a calculation of statistical power, but rather on an expectation of how many will be able to include over a project period of 12 months.

The study is anchored in the Psychiatric Center Glostrup, which has attached approx. 300 active patients divided into three teams. From previous experience with cognitive-behavioral therapy groups in OPUS, we know that at a given time it is possible to recruit a minimum of 10% of the total OPUS sample. Thus, it will be possible to continuously recruit a minimum of 30 patients for the project (of which 10 will be allocated to the intervention group and 10 to the control group). Against this background, we expect that - over a project period of 15 months - it will be possible to include approx. 60 patients divided into 30 patients in the intervention group and 30 in the control group.

**PROCEDURE FOR RECRUITMENT, INCLUSION AND RANDOMIZATION (GROUP ALLOCATION)**

Inclusion in the trial will be carried out by the trial leader. Patients who meet the inclusion criteria will be introduced to the trial by their contact person or a doctor in OPUS. Patients who are interested in knowing more about the trial will be given the participant information, including information about the lottery.

Written information about the project is sent/delivered at least 48 hours before the first appearance at OPUS Holstvej, 2nd floor, and information is given about the right to bring a companion.

On arrival, the patient is informed orally about the project by the project's research assistant and this answers any questions before the patient signs the consent statements. The patient is offered a reflection period of 24 hours after receiving oral and written information about the project.

The conversation will take place in a closed room, possibly together with the patient's companion. In the event of questions or complications, the patient will have the opportunity throughout the trial period to contact the trial manager or other healthcare professionals associated with OPUS Holstvej, 2nd floor, by telephone.

The patient is encouraged to bring a companion to the interview. During the interview, thorough information is given about the trial's background, purpose and content, as well as what it will entail to participate and what rights you have as a trial participant. Sufficient time will be given for the patient and any assistants can ask in-depth questions, and the interview will be adapted to the individual patient's prerequisites and needs. After the patient has signed informed consent, baseline measurements are carried out, including screening for rumination/concern, after which a draw is carried out. A draw is then made, which determines whether the patient must start the intervention immediately (the intervention group) or be entered on a waiting list, which is offered to start the intervention after approx. 12 weeks (waiting list control group). The randomization is done 1:1 in REDCap on the basis of an uploaded block randomization list (block size 4-6) generated by an external party. In addition, stratification is used to ensure equal distribution of gender and team affiliation between the groups. The patient is verbally informed about the result of the draw. Among the patients who are allocated to the intervention group, some will also be invited to take part in a qualitative interview.

**INTERVENTION**

The intervention consists of courses with group-based RFCBT described in a manual developed and published by Watkins [29] and translated for use in a Danish context by Morten Hvenegaard [30]. The course is expected to last approx. three months and is offered in connection with standard OPUS treatment (2-year intensive outpatient treatment offer) and begins with a group preparatory interview of approx. one hour duration followed by 11 group sessions of 2 hours duration once a week. The course ends with an individual interview (approx. one hour duration). The therapy includes review and completion of hand-outs, psychoeducation or dissemination of important messages from the manual, practical exercises and behavioral experiments (patients cooperate 2 and 2), guided visualization exercises as well as dialogue and exchange of experiences. The intervention takes place in OPUS and is overseen by an experienced psychologist (experiment leader) and a co-therapist.

Waiting list control group

Participants in the waiting list control group receive standard OPUS treatment and are offered the intervention after completion of follow-up measurements (expected 3 months).

**DATA COLLECTION METHODS AND OUTCOMES**

In order to answer the purpose of the survey and create the most well-informed basis for possible subsequent preparation of the protocol for a larger, randomized study, the trial combines quantitative and qualitative methods. This ensures not only an indication of the clinical relevance of the intervention, but also the organizational and individual prerequisites for implementation and potential benefit of the intervention.

**Quantitative methods and outcomes**

We will use quantitative measures in the form of standardized or patient-administered questionnaires and clinician-administered measurements to assess the intervention's potential clinical relevance and therapeutic effect. We have experience from previous trials regarding testing of mentally ill patients and are therefore confident that the following studies are realistic to carry out. Questionnaires and measurements are completed at baseline, and repeated after approx. 12 weeks (end of intervention). Follow-up clinician-administered measurements are made by a trained research assistant blinded to group allocation. We will also use quantitative metrics to determine recruitment and retention rates, attendance, resource usage and adverse events.

**Outcomes related to determining relevance**

• Worry/rumination behaviour:

o The frequency of rumination is measured using the Perseverative Thinking Questionnaire (PTQ). The PTQ is a self-reported questionnaire and includes 15 questions; each question is scored from 0 (never) to 4 (always mentioned) [32].

o Frequency of rumination in relation to depressive symptoms is measured using the Ruminative Response Scale (RRS). The RRS is a self-reported questionnaire and includes 22 questions; the total score expresses the severity of rumination [33].

**• Negative symptoms:**

o Negative symptoms will be assessed using the Brief Negative Symptoms Scale (BNSS) [34]. The scale includes six domains of negative symptoms: anhedonia, lack of normal emotional discomfort, asociality, avolition, dampened affect, and alogia; higher scores greater impairment/presence of negative symptoms.

**• Positive symptoms:**

o The presence of positive symptoms is assessed using the Scale for the assessment of positive symptoms (SAPS) [35]. The scale includes the subdomains hallucinations, delusions, bizarre behavior and linguistic (formal) thought disorder; each domain is scored 0-5, with five indicating higher difficulty.

• **Function level:**

o Level of functioning is measured using the Social Functioning Scale (SFS) questionnaire [36]. The SFS contains seven subscales: withdrawal/social engagement, interpersonal communication, independent-achievement, independent-competence, recreational, prosocial, and job/employment. Each part is scored 0 (never) to 3 (often), with higher scores indicating better functioning.

• **Depression:**

o Depressive symptoms are assessed using the Calgary depression scale (CDS) [37] in the form of a structured interview. The patient is scored 0-3 on a total of nine questions related to moodiness, hopelessness, self-deprecation, self-attributing notions of guilt, pathological guilt, morning depression, early morning awakening, suicide and observed depression; higher scores indicate higher severity of depressive symptoms. The scale has been validated for measuring depression in patients with schizophrenia [37].

• **Executive function:**

o Executive function is measured using the Behavior Rating Inventory of Executive Function (BRIEF) questionnaire [38] consisting of 75 statements that express function in the areas of impulse inhibition, flexibility, emotional control, self-monitoring, initiation, working memory, planning/organization and organization of Materials. Each statement is scored 1-3; higher scores indicate poorer functioning.

**Outcomes related to determination of feasibility**

• **Recruitment and retention rate**: We will record how many in the target group agree to participate in the experiment and compare this number with the number of possible (suitable) candidates. This achieves a percentage for recruitment (target 80%). In addition, we will record the individual's attendance at the therapy, and compare this with the number of possible therapy sessions during the period. This results in a retention percentage. Reasons for non-participation and any dropouts during the period (target max. 30%) are also explained qualitatively (see below).

**• Satisfaction with the intervention**: The aim is that 80% report satisfaction with the treatment.

**• Safety**: We will continuously record unintended events that may occur as a result of the intervention, including any worsening of symptoms (see also section on safety and ethics).

• Resource consumption: With a view to uncovering the sustainability of the intervention, we will uncover how many expenses (hourly consumption, transport, etc.) are associated with carrying out the intervention.

**Qualitative methods**

The value of qualitative research in the development and evaluation of complex interventions is widely recognized in psychiatry research [ 39 , 40 ]. It is planned to carry out individual semi-structured interviews with strategically selected participants allocated to the intervention group. The strategic selection is made with a view to ensuring maximum variation in relation to the criteria for inclusion. Selected participants are interviewed at baseline (focusing on motivation for participation) and again after 12 weeks (focusing on experience of participation). The information strength (saturation) is assessed after interviews with 10-12 participants and possibly a further 3-5 interviews are planned. For participants who dropped out of the intervention before the end of therapy, the reasons for this are carefully requested. In addition, a total of three semi-structured group interviews will be conducted (after 11 weeks) corresponding to the three intervention group courses, which are expected to take place within the project period. All interviews (individual and group) are conducted in a place, at a time and at a pace that feels natural to the participants.

**DATA ANALYSIS**

**Quantitative data**

Quantitative data is collected in RedCap. With regard to the choice of statistical tests, it applies to data where there will be continuous differences that these are analyzed using the paired t-test or a similar non-parametric test, while categorical data is analyzed using Pearson's chi-squared test . A two-sided significance level of p <0.05 is used. Continuous data are presented as mean ± standard deviation (SD).

**Qualitative data**

Qualitative data are recorded on a dictaphone and transcribed verbatim and then subjected to data-driven thematic analysis (editing style). Data from group interviews are analyzed using framework analysis [41], while data from individual interviews are analyzed using systematic text condensation or equivalent data-driven analysis [42]. All analyzes are carried out in collaboration between several researchers (researcher triangulation) and using NVivo software for qualitative data analysis. Any relevant theory (e.g. psychopathology, rumination) is included in the design of interview guides and discussion of the results of the analysis.

**SECURITY AND ETHICS**

Participants will be asked to give informed consent after oral and written information about the trial. The participants are informed that they can withdraw their consent at any time, without this having any effect on their treatment or association with OPUS in general. Trained and supervised by an experienced rater.

Risks, side effects and disadvantages

There is a risk that some participants may experience participation in the intervention as a stressful moment or, in the case of a lack of perceived progress, as a personal defeat. We will prevent this through continuous recognition of the individual's attendance. If a patient experiences deterioration (e.g. becomes suicidal), the trial leader will contact the doctor responsible for treatment in order to assess the need to implement relevant intervention measures (e.g. hospitalization). In addition, a decision will be made as to whether the patient can continue in the trial.

The project follows good clinical practice for monitoring suicide risk; including new onset or worsening of suicidal thoughts. This is assessed at all clinical meetings. If a patient experiences worsening and. e.g. expresses new or worsening suicidal thoughts, then the plan of action is for the experimenter, cf. the consent given at inclusion, to immediately contact the psychiatrist responsible for the treatment in order to assess the need to implement relevant intervention measures (e.g. intensification of the outpatient treatment or hospitalization). In addition, a decision will be made as to whether the patient must be excluded from the trial on the basis of the deterioration. Participation in the project is always voluntary, and consent can be withdrawn without this having any influence on the patient's rights or treatment.

If, during the trial, unexpected side effects occur of such a magnitude that it is considered unwarranted to continue the therapy, the trial will be stopped immediately. All possibly serious adverse events are reported to the Scientific Ethics Committee.

**Processing of sensitive personal data**

Storage and processing of sensitive personal information, including health information and information about purely private matters, will be reported to the Capital Region of Denmark's Knowledge Center for Data Reports. Cf. In accordance with the Data Protection Act and the Data Protection Regulation, information about the participants will only be collected to the extent that the individual consents to this. Physical media, such as consent forms, will be stored in a locked cabinet in a locked room at Psychiatric Center Glostrup. Electronic data will be stored in REDCap.

Information from the patient record In connection with a referral to the project from healthcare staff (treating doctor or contact person) at OPUS Holstvej, it may be necessary to pass on information from the patient's record in order to identify whether the patient can be included in the project (i.e. meets the inclusion criteria and does not meet exclusion criteria). The specific health conditions on which information is obtained from the medical record are primarily symptom level (duration and extent of positive and negative symptoms, comorbid psychiatric conditions, risk of suicide, current psychopharmacological treatment, number of hospitalizations). Possibly. control authority direct access to obtain relevant information in the patient's record in order to see information about the subject's health conditions, which is necessary as part of the implementation of the research project and for control purposes, including self-control, quality control and monitoring. All information collected in the project and from the journal will be used in the project in anonymized form.

**The benefits for patients**

The patients will go through an investigation program and have close personal contact with the trial managers. Furthermore, the patients who are randomized to the intervention group will be offered a specialized treatment for their rumination behaviour.

The treatment and any adverse events will be followed closely. The treatment is planned so that it follows current clinical guidelines.

The patients will have the opportunity to receive feedback on the examinations they undergo, which is why this will give the patients, and their therapists, further insight into the patient's individual symptoms and difficulties.

**The disadvantages for patients**

The disadvantages of the trial are assessed to be primarily linked to the time spent during the examinations (which are carried out at baseline and after the end of treatment). However, the time consumption is only approx. 1-2 hours per examination.

**The study's potential**

As stated in the background section, there is a need to develop effective interventions targeting rumination behaviour, negative symptoms and comorbid depressive symptoms in patients with psychotic disorders. There is currently only limited knowledge about the effect of rumination-focused therapy in this patient group, but there seems to be great potential for alleviating relevant symptoms and improving the patients' daily life. It is assessed by the research group that it is justifiable to carry out the trial and that the benefits clearly outweigh the few disadvantages that the patients could experience. The results of the trial will be able to give an indication of the effect of rumination-focused therapy, which can subsequently be sought to be confirmed in a randomized clinical trial regarding the possible preparation of well-documented, standardized guidelines for the treatment of this patient group, which can complement the current forms of intervention.

**Disclosure**

Both positive and negative and inconclusive research results will be published in international journals. The results will also be presented at national and international meetings and congresses.

The project will be registered on www.clinicaltrials.gov, once there is approval from the Scientific Ethics Committee and the Knowledge Center for Data Reviews, and before the first patient is included in the project.

**Compensation scheme**

The trial is covered by the patient reimbursement.

**ORGANIZATION AND FINANCING**

The study is anchored in OPUS, Psychiatric Center Glostrup, under the leadership of psychologist Christin Nymann Lund, who also handles the day-to-day and practical responsibility for carrying out the study, including the collection of quantitative data. The clinical responsibility for the examination, including assessment of any need for ongoing adjustment of inclusion and exclusion criteria, handled by specialist psychologist, MD, PhD. Louise Birkedal Glenthøj in collaboration with postdoc, psychologist Lars Clemmensen (VIRTU Research Group, Copenhagen Research Center on Mental Health, CORE). Qualitative studies are supervised by professor, psychologist Julie Midtgaard (Centre for Applied Research in Mental Health Care, CARMEN). The study is expected to be able to be completed without external funding.

# REFERENCER

1. van Os, J. and S. Kapur, *Schizophrenia.* Lancet, 2009. **374**(9690): p. 635-45.

2. Rangaswamy, T. and M. Greeshma, *Course and outcome of schizophrenia.* Int Rev Psychiatry, 2012. **24**(5): p. 417-22.

3. Jaaskelainen, E., et al., *A systematic review and meta-analysis of recovery in schizophrenia.* Schizophr Bull, 2013. **39**(6): p. 1296-306.

4. Vita, A., et al., *European Psychiatric Association guidance on treatment of cognitive impairment in schizophrenia.* Eur Psychiatry, 2022. **65**(1): p. e57.

5. Maggini, C. and A. Raballo, *Exploring depression in schizophrenia.* Eur Psychiatry, 2006. **21**(4): p. 227-32.

6. Hartley, S., C. Barrowclough, and G. Haddock, *Anxiety and depression in psychosis: a systematic review of associations with positive psychotic symptoms.* Acta Psychiatr Scand, 2013. **128**(5): p. 327-46.

7. Muller, J.E., et al., *Anxiety disorders and schizophrenia.* Curr Psychiatry Rep, 2004. **6**(4): p. 255-61.

8. Edwards, C.J., P. Garety, and A. Hardy, *The relationship between depressive symptoms and negative symptoms in people with non-affective psychosis: a meta-analysis.* Psychol Med, 2019. **49**(15): p. 2486-2498.

9. Temmingh, H. and D.J. Stein, *Anxiety in Patients with Schizophrenia: Epidemiology and Management.* CNS Drugs, 2015. **29**(10): p. 819-32.

10. Baynes, D., et al., *Depressive symptoms in stable chronic schizophrenia: prevalence and relationship to psychopathology and treatment.* Schizophr Res, 2000. **45**(1-2): p. 47-56.

11. Moritz, S., et al., *Do depressive symptoms predict paranoia or vice versa?* J Behav Ther Exp Psychiatry, 2017. **56**: p. 113-121.

12. Huppert, J.D. and T.E. Smith, *Anxiety and schizophrenia: the interaction of subtypes of anxiety and psychotic symptoms.* CNS Spectr, 2005. **10**(9): p. 721-31.

13. Bassett, M., D. Sperlinger, and D. Freeman, *Fear of madness and persecutory delusions: Preliminary investigation of a new scale.* Psychosis, 2009. **1**(1): p. 39-50.

14. Thomas, N., D. Ribaux, and L.J. Phillips, *Rumination, depressive symptoms and awareness of illness in schizophrenia.* Behav Cogn Psychother, 2014. **42**(2): p. 143-55.

15. Sellers, R., A. Wells, and A.P. Morrison, *Are experiences of psychosis associated with unhelpful metacognitive coping strategies? A systematic review of the evidence.* Clin Psychol Psychother, 2018. **25**(1): p. 31-49.

16. Cui, Y., et al., *Negative Schema and Rumination as Mediators of the Relationship Between Childhood Trauma and Recent Suicidal Ideation in Patients With Early Psychosis.* J Clin Psychiatry, 2019. **80**(3).

17. Hartley, S., et al., *An experience sampling study of worry and rumination in psychosis.* Psychol Med, 2014. **44**(8): p. 1605-14.

18. Badcock, J.C., G. Paulik, and M.T. Maybery, *The role of emotion regulation in auditory hallucinations.* Psychiatry Res, 2011. **185**(3): p. 303-8.

19. Vorontsova, N., P. Garety, and D. Freeman, *Cognitive factors maintaining persecutory delusions in psychosis: the contribution of depression.* J Abnorm Psychol, 2013. **122**(4): p. 1121-31.

20. Halari, R., et al., *Rumination and negative symptoms in schizophrenia.* J Nerv Ment Dis, 2009. **197**(9): p. 703-6.

21. Valmaggia, L.R., T.K. Bouman, and L. Schuurman, *Attention Training With Auditory Hallucinations: A Case Study.* Cognitive and Behavioral Practice, 2007. **14**(2): p. 127-133.

22. Ng, R.M.K., M. Cheung, and L. Suen, *Cognitive-behavioural therapy of psychosis: an overview and 3 case studies from Hong Kong.* Hong Kong Journal of Psychiatry, 2003. **13**: p. 26+.

23. Watkins, E. and S. Baracaia, *Why do people ruminate in dysphoric moods?* 2001, Elsevier Science: Netherlands. p. 723-734.

24. Lyubomirsky, S. and S. Nolen-Hoeksema, *Effects of self-focused rumination on negative thinking and interpersonal problem solving*. 1995, American Psychological Association: US. p. 176-190.

25. Lyubomirsky, S., N.D. Caldwell, and S. Nolen-Hoeksema, *Effects of ruminative and distracting responses to depressed mood on retrieval of autobiographical memories*. 1998, American Psychological Association: US. p. 166-177.

26. Nolen-Hoeksema, S., B.E. Wisco, and S. Lyubomirsky, *Rethinking Rumination.* Perspectives on Psychological Science, 2008. **3**: p. 400 - 424.

27. Nolen-Hoeksema, S., *The role of rumination in depressive disorders and mixed anxiety/depressive symptoms.* J Abnorm Psychol, 2000. **109**(3): p. 504-11.

28. Jones, N.P., G.J. Siegle, and M.E. Thase, *EFFECTS OF RUMINATION AND INITIAL SEVERITY ON REMISSION TO COGNITIVE THERAPY FOR DEPRESSION.* Cognit Ther Res, 2008. **32**(4).

29. Watkins, E.R., *Rumination-focused cognitive-behavioral therapy for depression*. Rumination-focused cognitive-behavioral therapy for depression. 2016, New York, NY, US: Guilford Press. xv, 360-xv, 360.

30. Hvenegaard, M., et al., *Group rumination-focused cognitive-behavioural therapy (CBT) v. group CBT for depression: phase II trial.* Psychol Med, 2020. **50**(1): p. 11-19.

31. Freeman, D., et al., *Effects of cognitive behaviour therapy for worry on persecutory delusions in patients with psychosis (WIT): a parallel, single-blind, randomised controlled trial with a mediation analysis.* Lancet Psychiatry, 2015. **2**(4): p. 305-13.

32. Ehring, T., et al., *The Perseverative Thinking Questionnaire (PTQ): validation of a content-independent measure of repetitive negative thinking.* J Behav Ther Exp Psychiatry, 2011. **42**(2): p. 225-32.

33. Treynor, W., R. Gonzalez, and S. Nolen-Hoeksema, *Rumination Reconsidered: A Psychometric Analysis.* Cognitive Therapy and Research, 2003. **27**(3): p. 247-259.

34. Mucci, A., et al., *A large European, multicenter, multinational validation study of the Brief Negative Symptom Scale.* European Neuropsychopharmacology, 2019. **29**(8): p. 947-959.

35. Andreasen, N.C., et al., *Correlational studies of the Scale for the Assessment of Negative Symptoms and the Scale for the Assessment of Positive Symptoms: an overview and update.* Psychopathology, 1995. **28**(1): p. 7-17.

36. Birchwood, M., et al., *The Social Functioning Scale. The development and validation of a new scale of social adjustment for use in family intervention programmes with schizophrenic patients.* Br J Psychiatry, 1990. **157**: p. 853-9.

37. Addington, D., J. Addington, and E. Maticka-Tyndale, *Assessing depression in schizophrenia: the Calgary Depression Scale.* Br J Psychiatry Suppl, 1993(22): p. 39-44.

38. Løvstad, M., et al., *Behavior Rating Inventory of Executive Function Adult Version in Patients with Neurological and Neuropsychiatric Conditions: Symptom Levels and Relationship to Emotional Distress.* J Int Neuropsychol Soc, 2016. **22**(6): p. 682-94.

39. Crawford, M.J., et al., *Evaluating new treatments in psychiatry: the potential value of combining qualitative and quantitative research methods.* International Review of Psychiatry, 2002. **14**(1): p. 6-11.

40. Whitley, R. and M. Crawford, *Qualitative research in psychiatry.* Can J Psychiatry, 2005. **50**(2): p. 108-14.

41. Rabiee, F., *Focus-group interview and data analysis.* Proc.Nutr.Soc., 2004. **63**(4): p. 655-660.

42. Malterud, K., *Systematic text condensation: a strategy for qualitative analysis.* Scand.J.Public Health, 2012. **40**(8): p. 795-805.
