# Supplementary material for: Study protocol for RUFUS—A randomized mixed methods pilot clinical trial investigating the relevance and feasibility of rumination-focused cognitive behavioral therapy in the treatment of patients with emergent psychosis spectrum disorders
Source: PLoS One. 2024 Jan 25;19(1):e0297118. doi: 10.1371/journal.pone.0297118 (PMC10810475; doi:10.1371/journal.pone.0297118)
Supplement: Attachment — Submitted filename: RESPONSE TO REVIEWERS DECEMBER.docx [file pone.0297118.s007.docx]

RESPONSE TO REVIEWERS DECEMBER

1. Multiple titles: On the title page, the title is “study protocol for RUFUS – a randomized mixed method pilot clinical trial investigating the relevance and feasibility of rumination-focused cognitive behavioral therapy in the treatment of patients with emergent psychosis spectrum disorders”. However, the title listed before the Introduction section is “study protocol for RUFUS … investigating feasibility, acceptability and potential efficacy …”. Please select one that more appropriately describes the study.

REPLY: Thank you for pointing out this inconsistency. We have changed it so it now reads “Study protocol for RUFUS - a randomized mixed methods pilot clinical trial investigating the relevance and feasibility of rumination-focused cognitive behavioral therapy in the treatment of patients with emergent psychosis spectrum disorders” both places.

2. Repetitive phrase in the aim and hypotheses section: it is currently written in the manuscript as “the aim of the current study is to investigate … potential indications of treatment effect ... as well as investigating potential indications of treatment efficacy”.

REPLY: Thank you for pointing this out. We have changed it to “The aim of the current study is to investigate feasibility, acceptability and potential indications of treatment effect of group RFCBT in the treatment of young people with psychosis spectrum disorders that display symptoms of rumination.”

3. Semi-structured group interviews: It remains unclear whether the authors meant 3 group interviews for the 30 participants in the intervention group (i.e., 10 participants in each of the 3 group interviews). Specifically, the notation of 3*n=10 is confusing since this indicates that the number of participants is not an integer number (i.e., 3.33).

REPLY: Thank you for bringing our attention to this unclarity. We have changed it in the section 2.6 Qualitative sub-studyso it now reads: “All participants in the intervention group are invited to a concluding group interview. Consequently, a total of three group interviews will be conducted, with a maximum of ten participants per group.”

4. Please spell out the whole name for RFCAT.

REPLY; This is a typo. It is meant to say RFCBT. We have changed RFCAT to RFCBT in section 3.1 Experimental group

5. Indirect questioning: it will be helpful to elaborate on what constitutes indirect questioning and how this will minimize the reporting bias.

REPLY: We have elaborated and added the following to the outcome section

"Indirect questioning refers to a method of asking questions that does not directly inquire about a specific piece of information but rather seeks to obtain that information indirectly. That is, instead of asking a straightforward or direct question, individuals use a more subtle or circuitous approach to gather the desired information. This approach is used to create a more comfortable atmosphere, minimizing the chances of biased or inaccurate responses due to the pressure of direct questions. By sidestepping direct queries, it helps foster honesty and reduces reporting bias, where the way questions are framed impacts the truthfulness of answers."
